# Aortic Aneurism

**Published:** 1870-03

**Authors:** F. J. VanVorhis

**Affiliations:** Stockwell, Indiana


Article IV. — *Aortic Aneurism — Pathological Specimen.* By F. J. VanVorhis, M.D., Stockwell, Indiana.

During the winter of 1868–9 I procured a subject for private dissection, in which there was an aneurism of the arch of the aorta of such unusual size as, in my judgment, to be worthy of record.

Subject was a man about 55 years old, 5 ft. 4 in. high, and weighed about 180 pounds.

The thorax was opened in presence of my student, Mr. J. T. McShane.

The aneurism was also seen and examined by Dr. M. Baker, and his students, Mr. Wells and Mr. Cowan.

The aorta was dilated from within one inch of the aortic valves to the middle of fifth dorsal vertebræ.

The following measurements will give some idea of size and shape of the aneurism:

Circumference of aorta, just outside of the pericardium, 8¾ inches. Innominata artery completely obliterated, and right subclavian and right common carotid separated 1¼ inches.

Circumference of aorta between these vessels, 9¼ inches; between right and left common carotid, 9 inches; between left common carotid and left subclavian, 8¼ inches; about the middle of descending part of arch of aorta, 6¼ inches.

Aneurism was ruptured one inch posterior to the origin of left subclavian.

As near as could be judged, the right ventricle was increased one-half in capacity, and its walls very thin and flabby. The capacity of left auricle was at least doubled, and its walls very thin. The left ventricle was considerably dilated. The walls were thinner than natural, and contained patches of calcareous matter, as did the coats of most of the arteries.

The right common carotid was two or three times its normal size. Femoral arteries so small as to appear almost impervious.

No history of the case could be obtained. The physician in charge of the case did not detect the aneurism, but supposed death occasioned by pneumonia. The want of a previous history deprives this case of all interest, except what belongs to a pathological curiosity.

